# Bioreactors, scaffolds and microcarriers and *in vitro* meat production—current obstacles and potential solutions

**DOI:** 10.3389/fnut.2023.1225233

**Published:** 2023-09-06

**Authors:** Magdalena Kulus, Maurycy Jankowski, Wiesława Kranc, Afsaneh Golkar Narenji, Maryam Farzaneh, Piotr Dzięgiel, Maciej Zabel, Paweł Antosik, Dorota Bukowska, Paul Mozdziak, Bartosz Kempisty

**Affiliations:** ^1^Department of Veterinary Surgery, Institute of Veterinary Medicine, Nicolaus Copernicus University in Toruń, Toruń, Poland; ^2^Department of Computer Science and Statistics, Poznan University of Medical Sciences, Poznan, Poland; ^3^Department of Histology and Embryology, Poznan University of Medical Sciences, Poznan, Poland; ^4^Department of Anatomy, Poznan University of Medical Sciences, Poznań, Poland; ^5^Prestage Department of Poultry Science, North Carolina State University, Raleigh, NC, United States; ^6^Fertility, Infertility and Perinatology Research Center, Ahvaz Jundishapur University of Medical Sciences, Ahvaz, Iran; ^7^Division of Histology and Embryology, Department of Human Morphology and Embryology, Wroclaw Medical University, Wroclaw, Poland; ^8^Division of Anatomy and Histology, University of Zielona Góra, Zielona Góra, Poland; ^9^Department of Basic and Preclinical Sciences, Institute of Veterinary Medicine, Nicolaus Copernicus University in Toruń, Toruń, Poland; ^10^Physiology Graduate Faculty, North Carolina State University, Raleigh, NC, United States; ^11^Division of Anatomy, Department of Human Morphology and Embryology, Wroclaw Medical University, Wroclaw, Poland; ^12^Department of Obstetrics and Gynecology, University Hospital and Masaryk University, Brno, Czechia

**Keywords:** bioreactor, cultured meat, scaffold, microcarriers, *in vitro* culture

## Abstract

*In vitro* meat production presents a potential viable alternative for meat consumption, which could provide the consumer with a product indistinguishable from the original, with very similar nutritional and culinary values. Indeed, the alternative products currently accessible often lack comparable nutritional value or culinary attributes to their animal-derived counterparts. This creates challenges for their global acceptance, particularly in countries where meat consumption holds cultural significance. However, while cultured meat research has been progressing rapidly in recent years, some significant obstacles still need to be overcome before its possible commercialization. Hence, this review summarizes the most current knowledge regarding the history of cultured meat, the currently used cell sources and methods used for the purpose of *in vitro* meat production, with particular focus on the role of bioreactors, scaffolds and microcarriers in overcoming the current obstacles. The authors put the potential microcarrier and scaffold-based solutions in a context, discussing the ways in which they can impact the way forward for the technology, including the use of considering the potential practical and societal barriers to implementing it as a viable food source worldwide.

## Introduction

Various product categories aim to replace conventional dietary meat. One category centers on plant or fungal substitutes, like soy or fungal protein, aiming to mimic animal product taste and texture. While this is the most developed option, achieving identical nutritional composition and indistinguishability remains a challenge (1). Insects are also considered as a cost-effective, eco-friendly meat source, but concerns about contamination, working conditions, and cultural resistance persist (2–4).

Lab-grown meat aims to use techniques of *in vitro* cell culture to obtain industrial quantities of tissues almost identical to animal meat (5). The ultimate goal is lab-grown meat that rivals traditional products in nutrition and taste, though replicating complex meat cuts is time-consuming and expensive. However, there are currently several obstacles to achieving this goal, such as increasing the scale of culture while maintaining the structure of the final product, which is why the researchers are now mostly focused on ground meat alternatives, characterized by simpler processes production and optimization (6). In producing cultured meat, diverse cell types like muscle satellite cells and stem cells are pivotal for growth and differentiation (7). Bioreactors play a crucial role in the meat production processes, offering controlled environments for large-scale cell cultures. These bioreactors come in different modes, such as batch, fed-batch, and continuous, facilitating various growth stages (8). All of these models present advantages and disadvantages, with different impacts on the process and outcome of *in vitro* meat culture. Moreover, scaffolds and microcarriers can enhance cell attachment, proliferation and differentiation, enabling larger-scale and more effective culture. However, while materials like chitin and cellulose showing promise in such application, there are also significant downsides of these materials that need to be considered before their implementation (9). Furthermore, in the recent years, technologies such as 3D bioprinting are also indicated as a solution to some of the obstacles of *in vitro* meat culture (10). While none of these technologies can be indicated as a on-fits-all solution to the obstacles associated with cultured meat production, understanding and optimizing them could potentially bring us closer to revolutionizing meat production in a sustainable and innovative manner.

As lab-grown meat presents a promising solution for sustainable protein supply and reduced environmental impact of animal husbandry, this review explores meat culture history, offers up-to-date insights into cell sources and *in vitro* meat production methods, and outlines its future prospects. Notably, the potential utilization of bioreactors, scaffolds, and microcarriers is discussed as a means to advance the technology and cut production costs.

## The history of cultured meat

While *in vitro* cultured meat may seem like a 21st century concept, it has been present in the imagination of artists and politicians for over 100 years. First mentioned by Frederick Edwin Smith, who predicted that it could 1 day be possible to produce an assortment of meat products using the source material obtained from a singular steak (8). This idea continued to be present in the literature of the 20th century, even appearing in the essay titled “Fifity Years Hence,” by the British prime minister Winston Churchill (1). Moreover, the scientific discoveries suggesting the possibility and viability of the idea of *ex vivo* meat production also had their beginning in the early 1900s. In 1912, Alexis Carrel managed to maintain a chick heart cell alive and beating *in vitro,* presenting a proof of concept of the possibility to culture animal tissues. However, despite the mentions of the topic in the literature, the cultured meat needed to be re-discovered by Willem van Eelen in the 1950s, who enriched and expanded the idea of laboratory meat production, laying foundations for the topic as we know it today (5). Another breakthrough came in 1971, the form of the first instance of muscle fiber cultivation, derived from the aorta of guinea pig by Russel Ross. In 1991 Jon F. Vein filed for and secured a patent for tissue-engineered meat production for human consumption, assuming production of muscle and fat tissues simultaneously to maintain the composition resembling animal meat. Furthermore, in 2001, a University of Amsterdam researchers also submitted a worldwide patent for an *in vitro* meat production process, using a collagen matrix seeded with muscle cells and suspended in medium (11). The first large investment in cultured meat research was made in a NASA-funded study, in which scientists cultivated muscle tissue from the common goldfish (*Carassius auratus*) in Petri dishes, with the goal of producing cultured animal muscle protein for long-term space voyages or habituation of space stations. The resulting cultured cells were garnished with spices evaluated by a test panel, which made the verdict that the product was of food quality (12).

Through the development of *in vitro* animal tissue culture technology, an increasing number of culture protocols are being developed, guaranteeing progress in regenerative medicine or pharmacology. It is advances in this field that make it possible to schedule the introduction of *in vitro* meat production (2, 13, 14). Discoveries in the early 2000s proved the existence of muscle stem cells, called satellite cells, which can organize themselves into muscle fibers (15, 16). By creating the first *in vitro* meat-based burger, researchers made a significant advancement in the field of *in vitro* meat production in 2013. A sensory panel in London’s Riverside Studios cooked and tasted a five-ounce cultured beef burger. The meat, which then cost more than $330,000, was grown in the lab using stem cells taken from a cow’s shoulder in just 3 months. The panelists agreed that the burger “nearly” tasted like a standard one. This fact, for the first time, allowed the general public to see lab-grown meat as a viable alternative for well-known and consumed meat products, raising expectations of the appearance of similar products on publicly available store shelfs in the relatively near future (12). Since then, there has been a surge in interest in using *in vitro* approaches to replace the production of meat from animals. This is a difficult task because the cultured meat must have a similar nutritional value as its animal-derived counterpart, as well as present similar flavor, texture, and look.

For future cultured meat to satisfy expectations, it must exhibit physical characteristics similar to traditional meat - appearance, flavor, aroma, texture. In addition to the muscle cells, other tissues such as fat, cartilage and connective tissue play an important role (13). It has been proposed that cultured meat could be the substrate for products such as sausages, burgers, nuggets etc.—a form of ground meat (17, 18). The products we consume must not only taste good and be of high quality. The appearance of a product is also important for the demand for it. In the case of cultured meat, the red color will have to be obtained through hemoglobin supplementation (19). There are various concepts for obtaining hemoglobin, which can be used as a substrate in the cultivation of muscle cells (20–22). The correct and acceptable perception of meat is linked to its olfactory qualities (23). Another important aspect is the fatty acid content (24).

The taste, smell and appearance of *in vitro* produced meat are very important. However, it is also necessary to develop the desired meat structure. Using the latest 3D printing techniques, it is possible to try to mimic the dense texture of real meat (25, 26). A comprehensive approach to meat production, with all its qualities, hygienic production and economic viability, is therefore a very complex and difficult issue, where interdisciplinary collaboration is essential.

While expenses associated with *in vitro* meat production decreased as the technology developed, with the current processes of meat production already significantly less costly than the original $330,000 burger. Nonetheless, the main issues still remain, with the requirement collect the necessary cells from live animals, the pricey, animal-derived serum used as a fundamental component of the growth medium for cell proliferation and differentiation, as well as the scalability of *in vitro* meat production process (27).

## Cells used for *in vitro* meat cultivation

Meat comprises around 90% muscle fibers, 10% fat, and less than 1% blood, with the numbers varying depending on the species of origin, cut type, as well as the age and diet of animals (7). Skeletal myocytes, together with adipocytes, fibroblasts, chondrocytes, and hematopoietic cell types, play a major supporting function in meat. For *in vitro* meat culture, the initial cell types must be able to self-renew to achieve sufficient numbers and differentiate into the mature cell lineages that make up the meat. Hence, the cultivation of muscle cells alone in the production of cultured meat is associated with unfavorable taste and textural qualities. To improve the condition of *in vitro* meat, cultivation should be based on cell co-cultures with adipocytes, chondrocytes, among others. Although difficulties are presented by different culture requirements, cell potential and also different substrate needs (28, 29). Adult stem cells and pluripotent stem cells are the two main types of stem types discussed in the context of cultured meat, with the former most commonly used in the currently run research and development related to that subject (5). Adult multipotent undifferentiated progenitor cells, found in particular tissues and organs of many animal species, have the capacity to develop into a variety of cell types, typically those appropriate for the organ or tissue in which they are found. The muscle tissue environment exhibits three main progenitor/stem cell types: muscle satellite cells, mesenchymal stem/stromal cells (MSCs), and fibro/adipogenic progenitors (FAPs). These progenitor cells can develop into skeletal myocytes, adipocytes, chondrocytes, and fibroblasts, among other important mature cell types. Located under the basement membrane of muscle fibers, muscle satellite cells are muscle-resident stem cells that have the potential to differentiate into myocytes, which then produce multinucleated myotubes that pack into myofibers. They are one of the most abundant tissue-specific adult stem cell populations (30), with well-established methods for of their isolation from live animals and *in vitro* culture (31). MSCs, on the other hand, can be found in a range of anatomical locations, but are more frequently derived from the bone marrow. MSCs have the capacity to differentiate into fibroblasts, adipocytes, chondrocytes and myogenic cells. Finally, FAPs are considered to comprise a distinct mesenchymal cell population that resides in the skeletal muscle’s interstitial region (32). Playing an important supporting role in the development and organization of muscles, they possess the ability for differentiation into fibroblasts and adipocytes, making up both connective and fat tissues making up meat (33).

While obtaining adult stem cells is relatively simple, and they technically possess the ability to differentiate into all the mature lineages making up the content of meat, there are still some significant limitations regarding their proliferation and maintenance *in vitro* (34). Furthermore, while most of the current studies regarding cultured meat rely on adult stem cells, it is still worth noting that pluripotent stem cells should not be outruled as a potential alternative. ESCs (embryonic stem cells) and iPSCs (induced pluripotent stem cells) are the most notable types of pluripotent stem cells, exhibiting high proliferative capacity in culture, and the ability to differentiate into a broad range of cell types derived from all the germ layers (mesoderm, endoderm, ectoderm). ESCs can be isolated from the part of the blastocyst known as the inner cell mass, formed during early embryonic development of mammals, while iPSCs are obtained by inducing adult somatic cells using a set of identified pluripotency factors (35). IPSCs are cells that are already differentiated but through various factors can be transformed into pluripotent cells (36). Such stabilized transfection stimulates embryonic gene expression patterns, leading to massive proliferation (37). The potential of these cells in the future has the possibility to be used in regenerative medicine (38).

**Figure 1:**
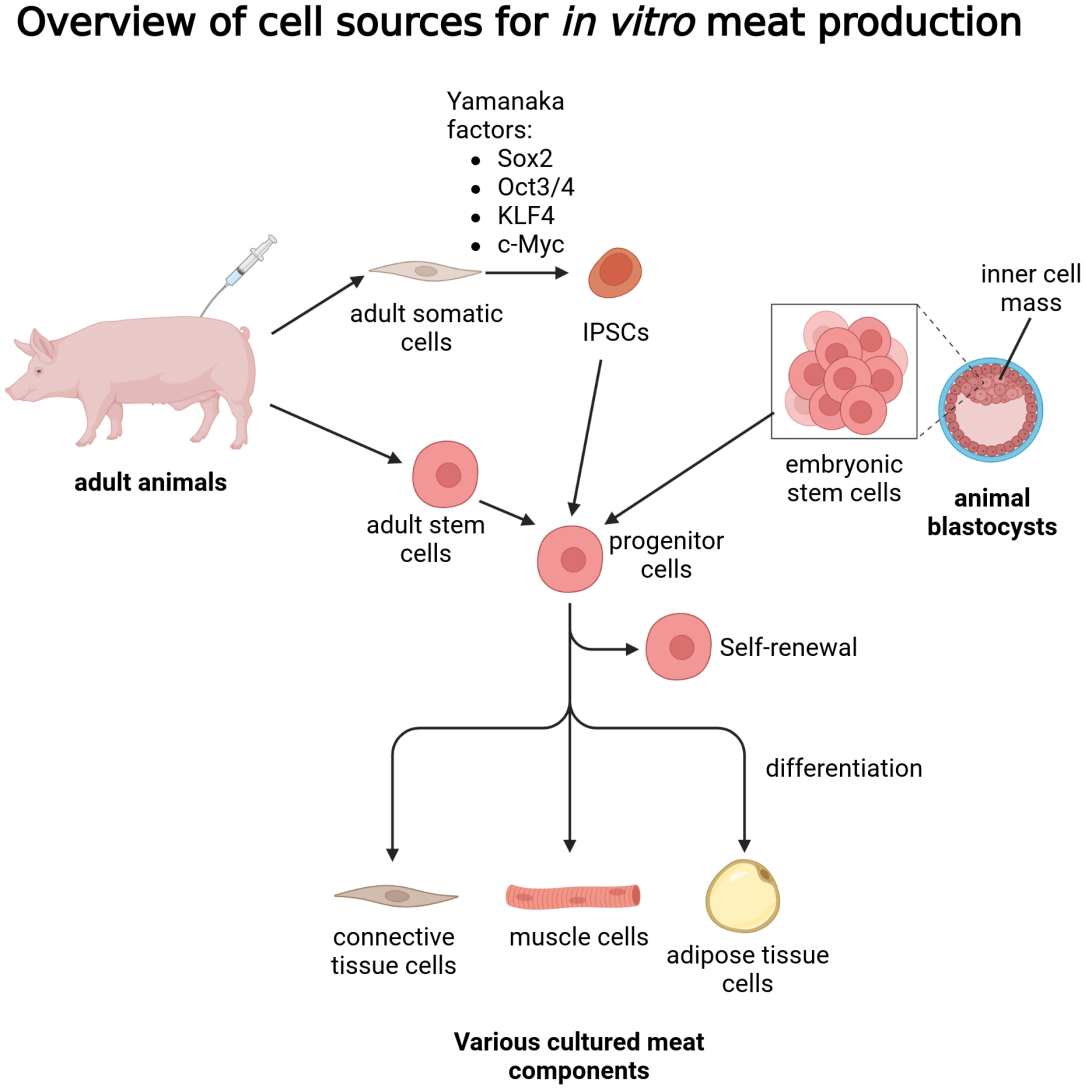
Overview of cell sources for *in vitro* meat production. Created with biorender.com.

While the topic of cell reprogramming has been already studied for a number of years, with significant breakthroughs related to human stem cells, there is still a notable gap in genetic and molecular knowledge regarding animals commonly used in the meat industry, with much more research necessary to employ iPSCs as a viable source of differentiated meat components (39). Additionally, there is still a lack of optimized isolation, culture and differentiation protocols for IPSCs derived from most commonly consumed animal species (40). In the case of ESCs, the relatively short time in which the blastocyst can be harvested, as well as some ethical concerns regarding their harvesting, effectively limit their use in the context of cultured meat (41). An overview of the above-described sources of cells with potential application in *in vitro* meat culture was summarized in Figure 1.

There have also been some studies regarding direct reprogramming of somatic cells into muscle progenitors, omitting the iPSC stage, through a process known as transdifferentiation. This approach is promising, as it allows to convert somatic cells directly into the lineage of interest, without the need to first dedifferentiate them into IPSCs, usually through overexpression of specific transcription factors. This mostly eliminates concerns regarding the remarkable plasticity of IPSCs, necessitating the use of a specific mix of factors to ensure their proper differentiation into muscle cells. Furthermore, it allows to mitigate the need for significant expansion of IPSCs *in vitro*, as, in theory, a larger number of somatic cells could be directly converted into muscle progenitors (42, 43). However, while more and more cells lineages have been recently obtained through these methods, it is still in relatively early development stages, requiring significantly more research before in could be applied in practice. Summarizing, adult stem cells are currently the population of choice for *in vitro* meat production, due to the ease of their isolation and differentiation, and relatively well-established culture protocols. However, they are limited in proliferation capacity, which sparks the interest into almost infinitely proliferating iPSCs. Nonetheless, before the latter can become a source of *in vitro* differentiated meat components there are several obstacles that need to be overcome, including significant cost of their induction and maintenance in culture, and low effectiveness and yield of induced differentiation (8).

Summarizing, obtaining cells for *in vitro* meat production will ensure a reduction in the number of animals needed for livestock production, as these cells can multiply many times (44). The development of stem cells from various tissues that can be cultured *in vitro* has been ongoing for many years. It is the refinement of culturing protocols that offers opportunities for the advancement of cultured meat production. Animal-derived adult stem cells or progenitor cells are the preferable source for *in vitro* meat production. Originating from fat, stem cells show promise due to their known transdifferentiation properties in the myogenic, osteogenic, adipogenic and chondrogenic directions (45). In addition, they have been shown to immortalize during long-term culture (46) and to differentiate into multipotent dedifferentiated fat (DFAT) cells and further into skeletal muscle cells (47).

An important aspect is to obtain a stable and homogeneous cell population for culture. Inducing different mutations by genetic or chemical engineering can lead to unlimited proliferation (48). This in turn can reduce the need for a continuous supply of fresh samples from donor animals. However, phenotypic drift and genetic instability continue to challenge the integrity of cultures in the laboratory (49). In addition, cultures face microbial infections. Embryonic, mesenchymal stem cells can be used to establish cultures (50), although satellite cells show great potential for differentiation and proliferation during meat production (51). In theory, cells in culture can proliferate continuously, but problems with accumulated mutations can lead to aging (52). Cell proliferation and differentiation are necessary for muscle cell cultures to function properly (53). The ability for long-term proliferation is influenced by the length of telomeres, a repeating sequence of guanine-rich at the end of chromosomes. With each successive round of replication, the chromosomes shorten. This concept, called the Hayflick limit, affects the limitation of each cell’s ability to divide (54). It is definitely a constraint that inhibits large-scale laboratory meat production. It is therefore necessary to look for opportunities to increase the regenerative potential. Regulating expression or adding telomerase (a telomere lengthening ribozyme) can effectively increase the proliferative potential of cells.

In addition to obtaining competent and proliferating cells, suitable culture media and stable conditions are essential for the production of cultured meat. When culturing mammalian cells *in vitro*, it is standard practice to add serum (55, 56) as it contains unique components (not only micro- and macroelements, but also hormones and growth factors) that promote proliferation and spread (57). However, serum is an expensive supplement and, in addition, its composition may not be reproducible and it can be a source of culture contamination (58). However, recent reports of the possibility of using disaccharides in protein-free cell culture are proving promising (59). The issue of serum in culture media is important in that cattle fetuses (fetal bovine serum, FBS) are most often used to obtain it. This in turn necessitates the use of animals, which was to be reduced with cultured meat. The serum also has to be controlled for sterility and supplemented, which also increases costs (60). There are alternatives to FBS in cell and tissue culture. Platelet lysates have been used successfully because a large proportion of the mitogenic agents in FBS are originated from activating platelets (61, 62). Although commercial media devoid of FBS are now available, research indicates that substances contained in FBS give the best results, and among substitutes, platelet lysate gives the best results (63).

## Bioreactors in *in vitro* meat culture

Products made from cultured muscle tissue, commonly referred to as cultured meat, are generated produced through the process of *in vitro* myogenesis. The process starts with sampling of muscle tissue from the animals of choice, followed by its dissociation and isolation of muscle stem cells. Next, the isolated stem cells are placed in a culture vessel, in which their initial growth and expansion occurs. Then, stem cells are differentiated into the mature muscle cells and matured, resulting in formation of artificial muscle tissue that is ready for harvesting and processing (64).

**Figure 2:**
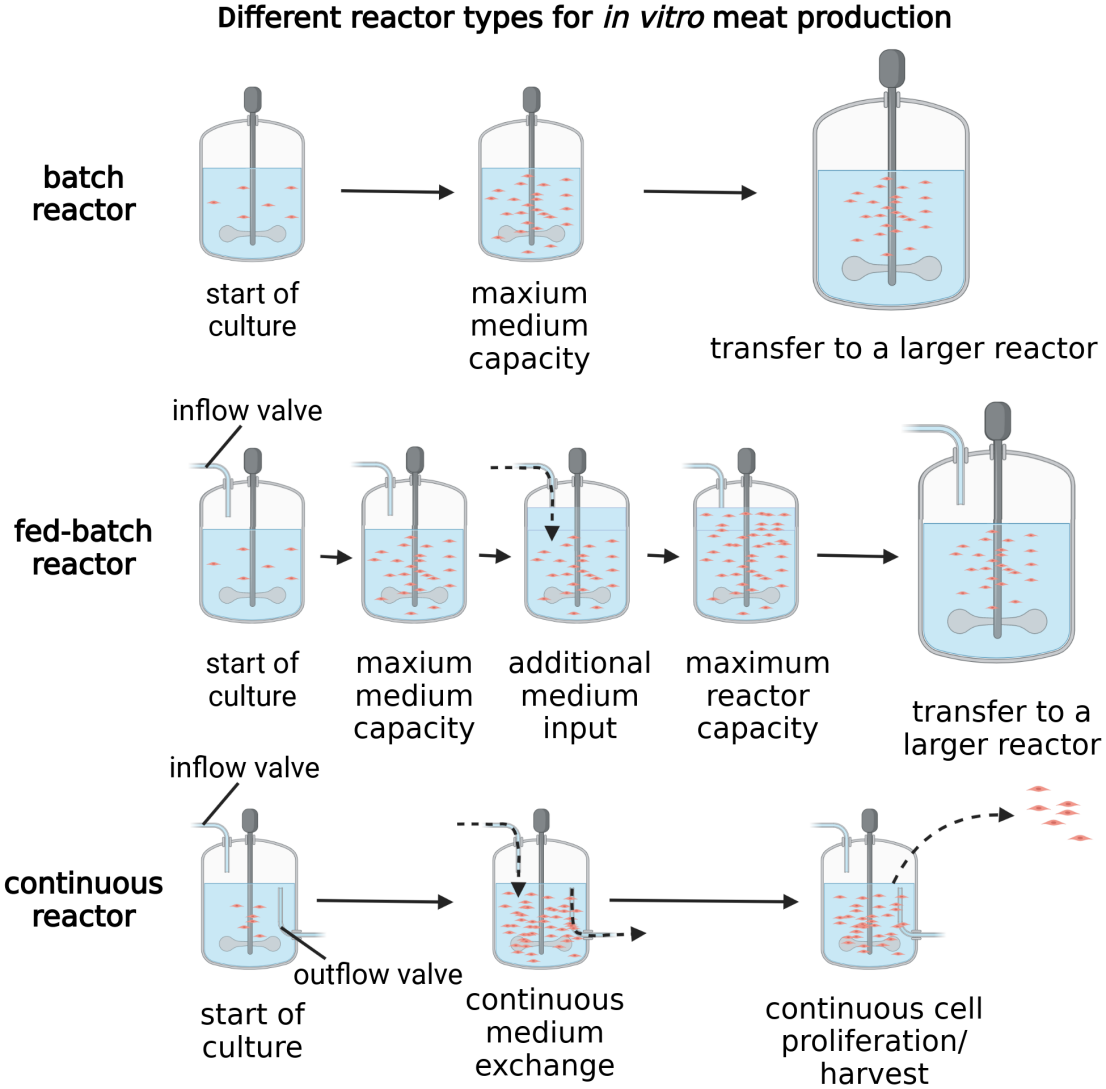
Different reactor types for *in vitro* meat production. Created with biorender.com.

All currently used meat culture approaches rely on bioreactors, a technology allowing to facilitate the large-scale culture necessary for *in vitro* meat production. Bioreactors serve a multitude of functions, from controlling the culture environment to maintain the biological conditions that facilitate effective proliferation and differentiation of cultured cells, to, in some cases, improving nutrient diffusion through the process of stirring or cell stimulation (8). Moreover, bioreactors are crucial to enable large scale cell culture and optimize medium uptake through its efficient recycling in the first steps of meat culture, highly reliant on rapid cell proliferation. Three main modes of bioreactors operation are currently used, with their classification based on the way in which the culture medium is administered to the culture: batch, fed-batch and continuous (Figure 2) (65). The first type, batch, contains a set medium volume, allowing the cells to proliferate until maximum density is reached, after which they need to be transferred into another, larger bioreactor (8). In turn, fed-batch, also known as semi-continuous bioreactors, allow for feeding of additional medium portions through a dedicated inlet, usually at set time intervals, calculated to achieve maximum proliferation. As spent medium outlet channel is not present in this reactor type, the culture volume increases over time, eventually reaching maximum capacity (66). This is the main difference between the fed-batch and continuous bioreactors, as the latter allows for constant medium administration at a set flow-rate, with simultaneous removal of the condition medium and cellular waste products (66). This reactor type should technically allow for the most optimized cellular growth rates, due to the maintenance of nutrient levels and cell numbers relatively constant. Furthermore, a subtype of continuous batch reactors, known as perfusion reactors, bases on the exchange of culture medium without disturbing the cellular contents of the reactors, which allows to achieve maximum medium recycling and vessel volume. However, this subtype is reliant on cell retention devices, ensuring the undisturbed proliferation and differentiation of cells during media exchange (67). Culture systems based on recirculation of cell culture supernatant (Alternating Tangential Flow; ATF, Tangential Flow Filtration; TFF), which differ from normal flow filtration, are also used in the context of perfusion reactors. They are designed to achieve cell retention and increase cell density (68). Promising methods of *in vitro* meat production, combining large scale cell culture, maximum automation and high efficiency of medium recycling, are mostly based on fed-batch or continuous type reactors (69).

Furthermore, bioreactors can also be classified based on the methods of content mixing or agitation, aiding the proliferation and differentiation of cells. The first type, mechanical bioreactors, use agitators or impellers to mechanically mix the culture volume. These bioreactors include the commonly used stirred tank reactor, which facilitates nutrient circulation and diffusion using impeller-based mixing. As for now, stirred tank bioreactors are most commonly used in large scale bioprocess, and might remain that way are amount of present know-how regarding their use in *in vitro* meat production. However, there are still some significant caveats of the stirred tank system, including relatively low scalability, potential turbulence resulting from impeller movement, or cell damage that might be caused by the moving parts inside the reactor (70). Current mammalian cell culture approaches are often based on a continuous type stirred tank reactor, in which this basic reactor concept is supplemented by continuous medium introduction (71). In another type of mechanical bioreactor, known as the rotating wall vessel reactor, the main vessel is spun around its central axis to allow for dynamic suspension culture of introduced cells (72). While this approach allows to eliminate the stress associated with internal moving parts, and stimulates cells to form 3D aggregates, it is often associated with increased early-culture apoptosis (73). The systems based on rotating-wall vessels are usually maintained in the batch culture approach, with the possibility to introduce a perfusion-based medium exchange to improve automation. The final, albeit rarely used, type of mechanical bioreactors is the so called mechanically active reactor system. This system is usually based on controlled dynamic compression of cells or scaffolds, aiding cellular development through *in vivo* environment simulation (74). As mechanical strength and alignment are important for the overall structure of the muscle tissue, this reactor type has could be useful in the context of *in vitro* meat production.

Another type of bioreactor uses liquid flow, instead of mechanical mixing, to achieve mixing of the vessel contents. This group of hydraulic bioreactors includes the hollow fiber bioreactor, that has already been used in several studies related to meat culture (75). In hollow fiber bioreactors, cells are seeded on a matrix of porous hollow fibers, allowing them to attach to its surface without disturbing the circulation of the medium. This approach is characterized by low mechanical stress and easier administration of particular nutrients, making it perfect for cells of high metabolism. Nonetheless, hollow fiber bioreactors are associated with a range of disadvantages, such as their relative complexity, limited scalability, proneness to clogging, limited oxygenation and relatively high cost (76). In a further bioreactor type, knows as the pneumatic reactor, mixing is conducted using a stream of gas instead of liquid. An example of such reactor, already used in *in vitro* meat culture, is the airlift bioreactor, utilizing the principle of gas–liquid circulation to create a gentle and continuous movement of the culture medium, promoting cell growth and enhancing mass transfer (77). Airlift bioreactors provide gentle and efficient mixing, minimizing shear stress on cells, allow for a relatively easy exchange of nutrients and gasses between the culture medium and cells, and are based on a fairly simple design, which makes their scalability comparable to other reactor types. However, some sources attribute this reactor types with too low mixing intensity and oxygen transfer rates, limiting their use in some types of cultured cells (78).

When designing a bioreactor system for the production of cultured meat, numerous issues should be taken into account from a cellular standpoint. The bioreactor might need to have a surface for cells to adhere, or it be able to support growing cells attached to scaffolds. This is due to the fact that a number of cell types found in meat, such as myocytes, are adhesion-dependent and need to be attached to a surface to effectively proliferate and differentiate. Furthermore, before the initial cell source differentiates into specific adherent cell types, it could require an initial population increase in suspension (8). In turn, non-adherent free-floating spheroid culture methods have the advantage of easier scalability and media delivery (79). Pluripotent stem cell sources, which can be cultivated as free-floating aggregates, would benefit more from this culture strategy, while other adult stem cell sources, such MSCs and muscle satellite cells, are easier grown in adherence-based cultures. The suitability of the aforementioned bioreactor types and stimulation techniques for particular cell sources should also be evaluated. For instance, many consider perfusion bioreactors as a potential strategy for producing specific *in vitro* meat products, as they combine continuous medium introduction with precise perfusion flow (18, 80). This allows the structure and size of the cultured tissue to be matched by adjusting the perfusion flow rate in these bioreactors. However, if scaffolds are used in perfusion bioreactors, with their size and scale linearly correlated with the perfusion flow rate, adjustment of flow could result in mechanical stress fall in pressure, potentially resulting in cell death. Summarizing, it is likely that some bioreactor systems are best for producing a particular sort of meat product *in vitro* and might not work well for producing meat in other forms or sizes. As the industry develops and strives to meet the demand for a wide variety of cultured meat products, it will be necessary to continuously optimize bioreactor systems for large-scale production.

## Scaffolds and microcarriers in cultured meat

Cells in their natural environment are in constant contact with each other, transmitting signals, metabolites and information - making life possible. In order to maintain this contact, a spatial relationship is essential, and the ECM is heavily involved in this process. In cell cultures, many cell types exhibit strong adherence properties, which is exploited in suspension cultures, on scaffolds or using microcarriers.

A scaffold is a biocompatible material, commonly used in tissue engineering, that can support the growth of adherent cells through mechanical support, facilitation of easier medium perfusion, and direction of tissue pattern formation (81). In the production of cultured meat, tissue scaffolds are often used to enhance cell differentiation and tissue development. However, many of the structural characteristics of the resulting meat result from the type and composition of these scaffolds (82). When it comes to the cell sources for cultured meat, the main contributions of scaffolds are to enable stem/progenitor cell differentiation via adhesion, and to affect the end product’s shape and cellular arrangement. Different types of scaffolds play a key role in the process of tissue formation and differentiation during the production of cultured meat. Typically, scaffolds used in cell cultures have a high affinity for cells (8). A key feature of scaffolds for cultured meat is to allow cell differentiation. In addition, due to the fact that the final product is edible, the materials used should be non-toxic and biodegradable (81, 82). As an alternative, the scaffold might be designed to dissolve or be eliminated before use (8).

In recent years, 3D culture techniques have developed very intensively. This cell culture system appears to be more efficient and the cells grow in an environment close to their physiological environment. Cells maintained in 3D culture are varied in terms of proliferation, differentiation and drug resistance (83, 84). As a result, during the proliferation differentiation stages of *in vitro* meat production, a scaffold is typically required, usually in the form of microcarriers, tiny, spherical beads consisting of either natural or synthetic biomaterials (85). To enable cell adherence to microcarriers, they are usually composed of extracellular matrix components or mimetic alternatives (86). The main goal is to produce or find materials that have characteristics similar to the extra cellular matrix, including similar cell adhesion mediation properties, facilitating efficient transduction of signals between cells, and providing similar levels of stiffness and/or elasticity of the produced “tissue” (87). Microcarriers are also used in bioreactor-based suspension cultures used in cultured meat production. Their high surface-area-to-volume ratio facilitates cell population scale-up by allowing a high-density cell adherence to the microcarrier (88). However, there is a problem with cell detachment from the microcarrier, which might be ineffective or result in cell death, reducing the yield of usable cells and tissues (89). Additionally, microcarriers can be incorporated into the cultured meat product, enabling the composition and characteristics of the microcarrier to be modified for enhanced flavor, color, and texture (90).

A further promising method to develop scaffolds for *in vitro* meat production is 3D bioprinting, which takes into account the distinctive architecture and diverse cellular makeup of various meat cuts. Using a computer-aided design model, 3D bioprinters work to create a 3D tissue structure one layer at a time by giving instructions on where to deposit a certain biomaterial (91). Bioink, a biomaterial deposited by 3D bioprinters, can be tailored to contain particular cells and biomolecules, allowing for simultaneous primary scaffold printing and supplementary element introduction. Three primary categories of 3D bioprinters exist nowadays: extrusion, laser, and inkjet. Thermal or piezoelectric technology is used by inkjet bioprinters to dispense bioink from their nozzles. Inkjet bioprinters have the advantage of being reasonably affordable, extremely accurate, and operate in moderate conditions that are unlikely to harm cells (92). However, when the size of the scaffold increases, the quality of 3D stacking is compromised by the low viscosity at which inkjet bioprinters discharge bioink, potentially leading to a less structurally robust scaffold (93). In turn, laser-based bioprinters print microscopic droplets using a laser beam and a proprietary lens. In addition to having higher resolution than other bioprinting modalities, laser bioprinters have the advantage of being nozzle-free, allowing for the printing of materials with greater viscosities. On the other hand, the method is difficult to scale up and the laser employed might unintentionally damage the cultured cells (94). Extrusion bioprinters, the last major type of 3D bioprinters, are manually or pneumatically propelled. The scaffold produced by this type of printers has robust mechanical qualities and enables the use of bioink with a very high viscosity. However, extrusion bioprinters produce mechanical stress at the nozzle tip, which restricts the types of biomaterials that may be utilized in the bioink and results in the lowest final scaffold resolution of the three types of bioprinters. The majority of products that can be produced using the current meat culture technologies, mostly composed of ground meat analogs, lack evident scaffolding architecture. This may be a reflection of the challenge of developing an edible scaffold that supports cell viability throughout differentiation and permits specific 3D cellular architecture in the finished product. While 3D bioprinting and microcarriers are promising means for the improvement of methods of *in vitro* meat culture, further technological advancement and purpose-fitting fine tuning of these technologies is required to successfully employ them in large scale protein fabrication (8).

Hydrogels are frequently used as scaffolding in current tissue engineering techniques because they may be specifically designed technology are required to produce scaffolds that address all of these factors simultaneously. For to mimic the 3D microenvironment suitable for specific cell types, as well as can be used as 3D bioprinting bioink (10). Another strategy to adapt currently available biomaterials for cultured meat production is to utilize biomaterials commonly used in the commercial food industry (95). Additionally, novel biomaterials are being investigated for the generation of cultured meat, with one example being plant-based scaffolds, which have an intrinsic biodegradable structure and endogenous proteins that contribute to the cell microenvironment (96).

One of the most common polymers found in nature is cellulose, which is the main component of plants. It shows great potential as a scaffolding material due to its versatility, biocompatibility. In addition, cellulose and its derivatives are functionalized by mixing them with other materials to improve their chemical, physical or biological properties (97, 98). A good solution is also the decellularization of, e.g., plant tissue with a detergent (e.g., SDS) in order to create pores in the plant cell membrane and release cellular components. Thanks to this process, it is possible to obtain a scaffold resembling a skeleton for muscle tissue, e.g., cardiac muscle (96). The decellularization process can be performed on any part of the plant, both the stalk and the leaves (99). The advantage of this type of scaffold is the ability to embed cells in such a way as to potentially mimic *in vivo* conditions in which cells occur without high financial outlays. Such solutions seem to be particularly useful in 3D cultures of musculoskeletal cells (99). Cellulose is found not only in the plant world, strains of bacteria such as *Acetobacter* spp. also produce cellulose. Bacterial cellulose, compared to plant cellulose, has a higher degree of polymerization, as well as a higher water-holding capacity. This type of scaffold is used as a gelling agent, stabilizer and thickener. In clean meat production, it is used to give juiciness (100).

Another equally popular substance that can be used as a scaffold is chitin, a polysaccharide found in the exoskeletons of arthropods and cell walls of fungi. It presents certain advantages as a scaffold material, as its fibrous structure provides mechanical support and mimics the texture and structure of muscle tissue. Chitin is also mostly biocompatible and can support the attachment, growth, and differentiation of cells involved in meat tissue regeneration (9). Furthermore, it is widely available in the form of waste from the seafood industry, such as shrimp shells and crab shells. Utilizing such waste material for *in vitro* meat production can reduce waste and contribute to a more sustainable approach. Nonetheless, there are also several disadvantages to its use, mostly related to challenges associated with its processing, due to its limited solubility in most common solvents, and the potential presence of scaffolds remnants in the final meat products, that could impact its texture and taste (101, 102).

Natural biomaterials used as scaffolds have many advantages, such as: high biocompatibility and low immunogenicity. These properties are important, as they allow to limit the stress exerted on cultured cells, allowing to minimize the impact of culture conditions on proliferation and differentiation rates. Furthermore, they are often biodegradable, which is advantageous if they are not to be present in the final cultured meat product (103). However, they also present some limitations. Natural biomaterials sourced from biological origins may exhibit inherent variability in their composition and properties. This variability can affect their mechanical strength, degradation rate, and other characteristics, making it challenging to achieve consistent scaffold properties. Furthermore, compared to synthetic biomaterials, natural biomaterials often have inferior mechanical properties, such as lower tensile strength or elasticity. This limitation can restrict their use in load-bearing applications or areas that require high mechanical stability. Finally, natural biomaterials are typically obtained from biological sources, which can limit the control over their structure, porosity, and other physical properties. Achieving precise scaffold architecture and pore size distribution may be more challenging compared to synthetic biomaterials (104).

Summarizing, capitalizing on the advantages of various material sources and production techniques of scaffolds and microcarriers could allow the expansion of the range of potential cultured meat products, as well as overcoming of the challenge of production scale increase. However, the notable disadvantages of the presented material types, as well as the caveats of the currently used fabrication and processing techniques need to be taken into account, to mitigate their impact on the final product, and allow to select the right scaffold or microcarrier for particular *in vitro* meat culture-related application (103, 105, 106).

## The future of *in vitro* meat

If the concept of cultured meat will develop further in the near future, it will potentially be able to offer adjustment of the biochemical composition of meat, through metabolic engineering. It has been shown that it is possible to produce bioactive metabolites of plant origin, which affects the functionality of food. Antioxidant factors such as phytoene carotenoids, lycopenes and beta-carotene could be produced by modified bovine and mouse muscle cells (107). Furthermore, assuming that the introduction of cultured meat into commercial use is, for the time being, quite remote, the use of this vertebrate protein as a therapeutic supplement should be considered. Such a product could contain specialized therapeutic substrates, in which case appearance or texture and high production costs would not be such an important consideration (5).

The mere fact that it is possible to produce meat *in vitro* that is fit for consumption is just the beginning of complex mass production. The next step in the future production of vertebrate proteins will be the differentiation of this product into various types of meat. When analyzing eating habits in many cultures and countries, one must consider the consumption of meat from different parts of the animal body. Research is now focusing on creating patterns of proportions of major components in different parts, which can be applied to future production (108, 109).

The undeniable advantage of commercially used cultured meat, in the future, would be the possibility to eat meat products while minimizing the reliance on animal farming (6). An additional important aspect is the potential qualitative monitoring of the cultured meat in terms of composition, flavor modifications, fatty acid composition or the ratio of saturated to unsaturated fatty acids (110). Future developments in the commercialization of cultured meat should focus on rapid and profitable production. Concentrating energy and time on culturing muscle alone (without loss to the development of other organs, skeleton) can be beneficial from the perspective of cost and waste efficiency (44). In addition, compared to the years it takes to grow, e.g., mature cattle, obtaining muscle fragments only in a short period of time seems advantageous. Furthermore, lowering the reliance on industrial farming could significantly shrink the land area currently used for livestock production, as well as lower the dependence on natural resources. Reducing large-scale transport could also limit environmental pollution, as the production of cultured meat can be carried out in multiple locations to ensure continuous access. As a result, expansion of the *in vitro* cultured meat sector could allow for maintenance of some extent of meat product consumption, while reducing pollution and environmental degradation, further decreasing animal suffering, as well as potentially lowering the chance of antibiotic resistance and zoonotic diseases (111).

However, the challenges to mass production are the undeniably high costs, generation of suitable stem cells, appropriate media, the industrial-scale tasks of tissue engineering and general consumer acceptance (13). Moreover, while the benefits that cultured meat could bring are appreciated, there is concern that the production of this protein will be profit-driven and dominated by big companies (112). Future consumers of cultured meat will need to be convinced of its benefits, as their positive attitude and awareness of the sustainability of its production will be crucial to market such products (113). Most notably, the current disadvantages of cultured meat, such as high cost, production inefficiency, and a lack of ability to mimic more complex meat products will need to be solved before cultured meat products will be considered as a viable alternative by the general public.

## Conclusion

Most of the developed countries are aware of problems of environmental degradation, diseases, antibiotic resistance will try to find solutions and introduce some restrictions regarding the meat industry. Nonetheless, many of the currently available meat alternatives are characterized with some notable caveats. The production of cultured meat is a promising future prospect, that could potentially mitigate the need of meat industry expansion resulting from population growth. The technology is rapidly developing, and the first cultured meat products are seemingly ready for commercialization.

Achieving high-quality cultured meat involves using a variety of cell types, including stem cells like muscle satellite cells, MSCs, and FAPs, to ensure desired taste and texture. Bioreactors play a pivotal role in enabling large-scale production, offering different modes for controlling culture environments and nutrient supply. Scaffolds and microcarriers are essential for facilitating cell attachment and growth, with 3D bioprinting and microcarriers offering potential for creating complex tissue structures. However, challenges remain in bridging knowledge gaps and optimizing stem cell protocols, while selecting suitable bioreactor systems is crucial. Despite these hurdles, cultured meat holds promise for addressing sustainability and ethical concerns in conventional livestock farming, making ongoing research imperative for realizing a more sustainable food industry.

## Author contributions

PM and BK contributed to conception and design of the review. MK, MJ, and WK wrote the first draft of the manuscript. AG and MF wrote sections of the manuscript. PA, DB, PD, and MZ contributed to the review and revision of the manuscript. All authors contributed to the article and approved the submitted version.

## Funding

This research was partially supported by the Cultured Meat Enhancement Fund.

## Conflict of interest

The authors declare that the research was conducted in the absence of any commercial or financial relationships that could be construed as a potential conflict of interest.

## Publisher’s note

All claims expressed in this article are solely those of the authors and do not necessarily represent those of their affiliated organizations, or those of the publisher, the editors and the reviewers. Any product that may be evaluated in this article, or claim that may be made by its manufacturer, is not guaranteed or endorsed by the publisher.
